# Comprehensive Analysis of Innate Immunophenotyping Based on Immune Score Predicting Immune Alterations and Prognosis in Breast Cancer Patients

**DOI:** 10.3390/genes13010088

**Published:** 2021-12-29

**Authors:** Weiguang Liu, Lingling Xia, Zhengmiao Xia, Liming Chen

**Affiliations:** Department of Biochemistry, School of Life Sciences, Nanjing Normal University, Nanjing 210023, China; 171201005@njnu.edu.cn (W.L.); xia8242021@163.com (L.X.); skxzm2019@163.com (Z.X.)

**Keywords:** breast cancer, innate immune, immune microenvironment, checkpoint immunotherapy, small molecular target drug

## Abstract

Breast cancer is the most common cancer, with the highest mortality rate and the most diagnosed cancer type in women worldwide. To identify the effect innate immune checkpoint for breast cancer immunotherapy, the innate immune prognostic biomarkers were selected through the ICI score model and the risk model in breast cancer patients. Moreover, the reliability and accuracy of the ICI score model and the risk model were further examined through the analysis of breast cancer prognosis and immune cell infiltration. The pan cancer analysis further confirmed and selected CXCL9 as the key innate immune checkpoint for breast cancer immunotherapy and identified three small molecular drugs for target CXCL9 through molecular docking analysis. In summary, CXCL9 significantly correlated with the prognostic of breast cancer and immune cell infiltration and could be innate immune checkpoint for breast cancer immunotherapy.

## 1. Introduction

Breast cancer is the most common cancer, with the highest mortality rate and the most diagnosed cancer type in women worldwide [1]. Based on the clinical characteristics and gene expression, breast cancer is a heterogeneous disease with multiple molecular subtypes, including ER+, HER2+, and triple-negative [2,3,4]. Among these subtype breast cancer patients, the treatment strategies and clinical characterization vary differently [5]. As so far, the treatment solutions of breast cancer patients include surgery therapy, radiation therapy, chemotherapy, and hormone therapy [6]. Even though the treatment options for breast cancer patients have been more mature, the high fatality rate of breast cancer has not been effectively improved. Therefore, novel therapy routes for breast cancer patients are urgently needed. In recent research, immunotherapy has gradually become an emerging effective treatment method for multiple type of cancer, including breast cancer and subtypes of breast cancer [7,8]. However, the effective therapeutic target and specific treatment strategy for breast cancer immunotherapy still needs further study.

Recently, the new upsurge of tumor research from tumor immunity and tumor microenvironment were stimulated, and it is believed that tumor immunity will be the most powerful weapon to overcome cancer treatment [9,10,11]. With the progress of cancer immunotherapy, the importance of innate immune systems in antitumor were gradually attracted the attention of researchers [12,13,14,15,16]. Innate immune response as the first line and connect to adaptive immunity for protect the human body stay away from pathogens and tumors through multi-type special immune cells and pathways. There are various types of cells involved in innate immunity, including innate lymphoid cells (ILCs), macrophages and natural killer (NK) cells. More and more research evidence suggested that most of the immune cells in and around solid tumors come from the innate immune system in multiple type of cancer, including breast cancer [17,18,19,20,21]. Moreover, the importance of innate immune in cancer immunology and anticancer progress were further studied. Oncolytic herpes simplex virus-1 [16,22] significantly promoted the infiltration and activation of innate immune cell in tumor microenvironment via mediated tumor lysis. The high expression level of TIM-3 from tumor-associated dendritic cells significantly decreased innate immune response through its interact with HMGB1 and suppressed the recognition and recruitment of the molecular of nucleic acids [23]. These results suggested that the key role of innate immune response in the progress of tumors and may be the core element of the success of cancer immunotherapy. Furthermore, the treatment of immune checkpoints for cancer immunotherapy significantly inhibited antitumor immunity and innate immune cells mediated immune responses, including CTLA-4 [24,25,26], PD-1 [27,28,29] and TIGIT [30,31,32]. Although the research study of innate immune response in cancer has made great progress, the effect innate immune checkpoints for cancer immunotherapy based on innate immune response systems still needs further exploration. 

To understanding and screening the specific innate immune checkpoints for breast cancer immunotherapy, a series of bioinformatics analysis were performed for the transcriptome profiles of innate immune response genes in breast cancer patients via TCGA datasets and GEO datasets. In this study, we constructed and calculated innate-cluster-immune (ICI) score to predict the overall survival and prognosis of breast cancer patients through the expression level of significantly differentially expressed innate immune response prognostic signatures. Besides, the model of risk prognostic was also constructed. Comprehensive analysis of the effectiveness of risk score and ICI score, 11 candidates innate immune response signatures were selected. Pan analysis for those 11 candidates innate immune response signatures and found that CXCL9 may be the key innate immune checkpoint for breast cancer therapy.

## 2. Materials and Methods

### 2.1. Data Download and Preprocessing

The normalized transcriptional expression profile of breast cancer patients of were download from TCGA database. A total of 1109 breast cancer patients’ tissues and 113 normal breast tissues were included from TCGA-BRCA project. Besides, the mRNA expression data of 17 normal breast tissue samples and 104 breast tumor tissues were obtained from GEO GSE42568 project. To analyze innate immune checkpoint for breast cancer patients, we downloaded the genetic information of 1378 genes related to innate immunity from the InnateDB database. The significantly differentially expressed immune-related genes in breast cancer tissues compared with normal breast tissues were identified (|logFC (fold change)| >1 and FDR value < 0.05) with R packages edgeR [33] and limma [34].

### 2.2. Identification of the Innate Immune-Related Signatures

Kaplan–Meier survival analysis was performed through the survival package to screen differentially expressed innate immune-related genes (IIRGs) with *p*-value < 0.05 as prognostic signatures to predict breast cancer prognosis. A total of 20 IIRGs were selected for breast cancer. Furthermore, CNV analysis were performed for those 20 IIRGs in breast cancer patients group compared with normal group.

### 2.3. Consensus Clustering Analysis

To further understand the correlation between 20 innate immune-related signatures and the innate immune subtype of breast cancer, we clustered the expression profiles of breast cancer patients into three subtypes through the ConsensusClusterPlus package [35]. Then, overall survival analysis were performed among three innate immune subtype of breast cancer patients via chisq. test. Besides, we also performed GSVA analysis for three innate immune subtype of breast cancer patients through the R package, GSVA [36].

### 2.4. Functional Enrichment Analysis and Gene Set Enrichment Analysis

Firstly, the differentially expressed analysis among three innate immune subtype of breast cancer patients. Secondly, Gene Ontology (GO) enrichment analysis for differentially expressed gene sets through using the R package clusterProfiler (FDR value < 0.05) [37]. Gene Set Enrichment Analysis (GSEA) is a computational method to identify whether the expression of the specific gene sets show statistically significant differences between two groups. We performed GSEA analysis and multiple GSEA analysis in immune response pathway via The Molecular Signatures Database (MSigDB).

### 2.5. Constructed and Calculated the Innate-Cluster-Immune (ICI) Score

Next, based on the expression profiles of three innate immune subtype of breast cancer patients, we constructed and calculated the (innate cluster immune) ICI score via Principal component analysis (PCA) method through using R package Boruta [38]. Then, overall survival analysis was performed between the high ICI score group and the low ICI group in breast cancer patients. Besides, TMB analysis was performed between the high ICI score group and the low ICI group. Overall survival analysis for TMB score and ICI score was combined with TMB score in breast cancer patients.

### 2.6. Establishment and Validation of the Risk Prognosis Model 

Risk prognosis model was established based-on the innate immune-related prognostic signatures by the LASSO Cox regression and multivariate Cox regression analysis through R packages glmnet [39] and survival. Then, breast cancer patients were divided into high-risk group and low-risk group according to the risk prognosis score. Kaplan–Meier survival analysis was performed between high-risk group and low-risk group. Then, based-on the risk score and clinical indicators (Age, T, Stage, M, N), the plot of prognostic nomogram was established through multivariate regression via R packages survival and regplot. And the correlationship of clinical indicators and risk prognosis score were assessed between high-risk score group and low risk score group in breast cancer patients, including age, stage, T, M and N. Besides, the correlationship of the risk score and the expression of immune infiltration pathway, m6A readers and immune checkpoint in breast cancer patients were evaluated. 

### 2.7. Statistical Analysis

All statistical analysis and most of the bioinformatics analysis were performed via R (R version 4.0.2) packages including RNAseq count data normalization, differential gene expression analysis, Kaplan–Meier survival analysis, cox regression analysis and LASSO regression analysis. Heatmap was performed by pheatmap package. Kaplan Meier survival curves were plotted via survival packages. *p*-value < 0.05 was considered to be statistically significant.

## 3. Results

### 3.1. Identification of the Innate Immune-Related Prognostic Signatures in Breast Cancer Patients

**Figure 1:**
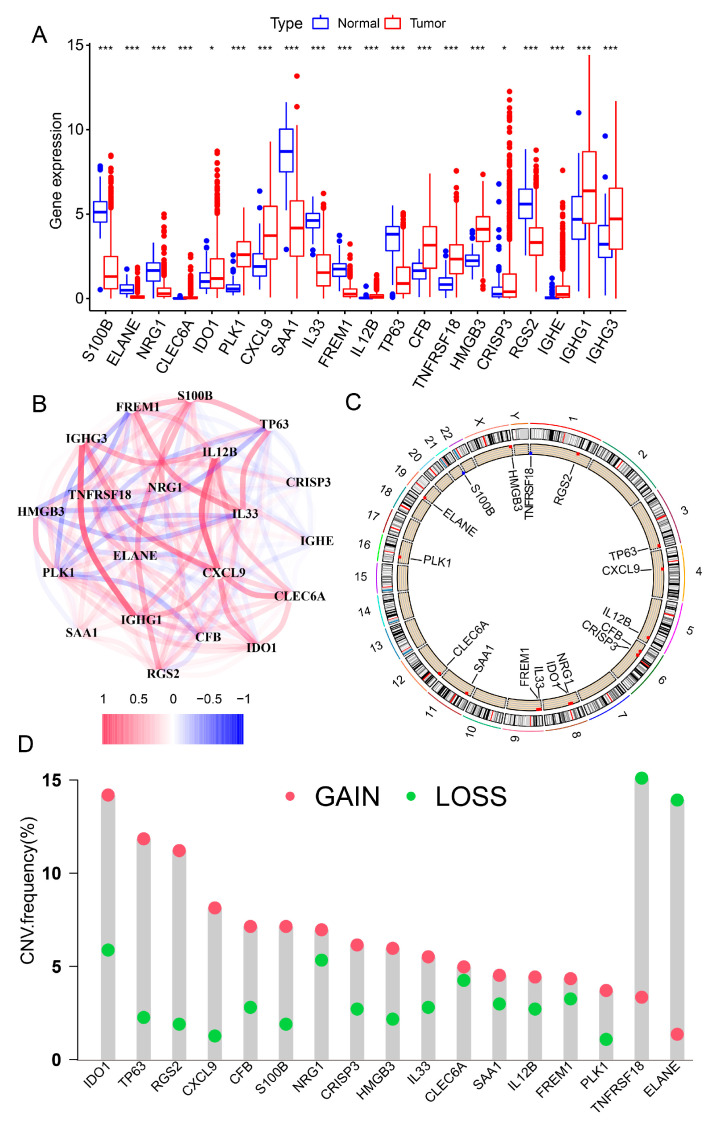
Identification of differentially expressed innate immune-related signatures in breast cancer patients. (**A**) The expression level of these innate immune related signatures in breast cancer were exhibited via the boxplot. * *p*  <  0.05, *** *p*  <  0.001, NS for not significant (*t*-test). (**B**) Based-on the transcriptome profiles in breast cancer, the network of the correlation of these innate immune-related signatures were analyzed through correlation analysis. The CNV frequency of immune related signatures were shown in (**C**,**D**).

Firstly, we downloaded the expression profiles and the clinicopathological characteristics of breast cancer patients from The Cancer Genome Atlas Program (TCGA-BRCA) and Gene Expression Omnibus (GEO) database (GSE42568). Then, we identified 103 innate immune-related genes were significantly differentially expressed in breast cancer tissue samples compared with normal tissue samples (FDR < 0.05, |logFC| > 2; Figure S1A). Secondly, to further screening prognostic signatures, univariate Cox regression analysis were performed and the results found that 199 innate immune-related genes were selected. Analysis of the transcriptome profiles and the results of the univariate cox regression analysis found that 20 out of these 103 differentially expressed innate immune-related genes could be mapped to the prognostic signatures in breast cancer patients (Figure S1B). The expression levels of these 20 innate immune-related prognostic signatures in breast cancer were shown in Figure 1A. And these prognostic signatures all significantly differentially expressed between tumor group and normal group. To further understanding the importance of these 20 innate immune-related prognostic signatures in the progress of breast cancer, the correlation of these 20 innate immune-related prognostic signatures were analyzed and exhibited significantly strongly correlation in breast cancer patients (Figure 1B). As we all known, copy number variations (CNV) is one of the mainly factors to affect the expression abundance of genes in multiple cancers [40,41,42]. Thus, we performed CNV frequency analysis for those 20 innate immune-related prognostic signatures in breast cancer. It’s noted that 17 out of 20 innate immune-related prognostic signatures were shown differentially levels of the CNV of gain and loss in breast cancer patients (Figure 1C,D). Besides, the mutation level of those 20 innate immune-related prognostic signatures also detected through R packages maftools and shown in Figure S1C. Furthermore, we further confirmed that 15 out 20 innate immune-related prognostic signatures exhibited significantly differences overall survival probability between the high gene expressed group and the low gene expressed group in breast cancer patients (Figure S2A–O).

### 3.2. Construction of the InnateImmCluster Molecular Subtypes Based on the Expression of Innate Immune-Related Prognostic Signatures in Breast Cancer

**Figure 2:**
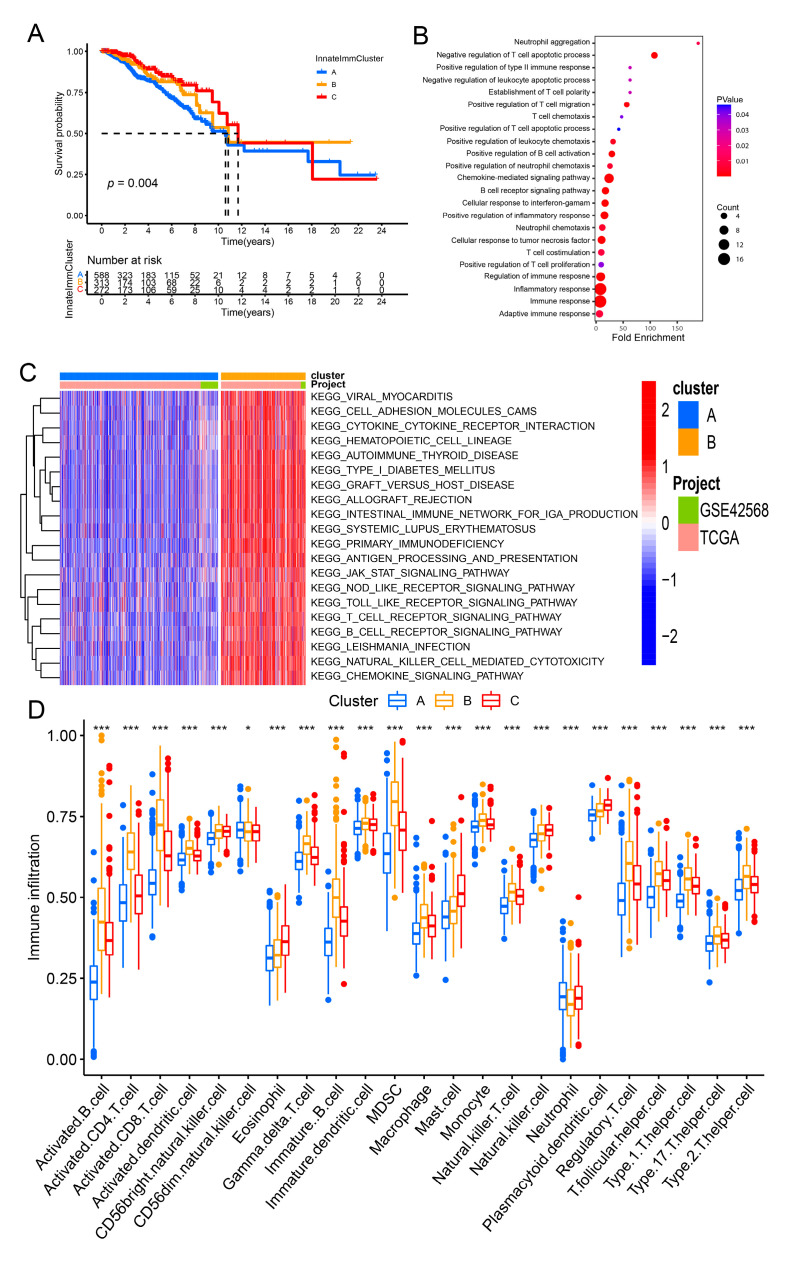
Identification of the InnateImmCluster molecular subtype of breast cancer by the expression of innate immune-related signatures in breast cancer. (**A**) Overall survival of breast cancer patients were analyzed among the three cluster of InnateImmCluster molecular subtype. (**B**) GO enrichment analysis for differentially expressed genes among the three cluster of InnateImmCluster molecular subtype in breast cancer. (**C**) GSVA analysis revealed that most of the immune response related pathway were significantly repressed in cluster A of InnateImmCluster molecular subtype in breast cancer compared with cluster B. Boxplot showing that the abundant of immune cells infiltration (**D**) among these three clusters of InnateImmCluster molecular subtype in breast cancer patients. * *p* < 0.05, *** *p* < 0.001, NS for not significant (Chi-Squared test).

Then, identification of three innateImmCluster molecular subtypes via the expression levels of 15 innate immune-related prognostic signatures in breast cancer through R packages, ConsensusClusterPlus (Figure S3A). And Kaplan–Meier survival analysis suggested that significantly differences in overall survival probability of breast cancer among these three-breast cancer innateImmCluster subtype (Figure 2A). Then, the GO enrichment analysis and KEGG pathway enrichment analysis was performed for differentially expressed genes among these three innateImmCluster molecular subtypes in breast cancer patients. These differentially expressed genes were significantly enriched mainly involved in immune-related pathways, including T cell chemotaxis, regulation of B cell activation, inflammatory response and TLR signaling pathway (Figure 2B and Figure S3B). To further confirm the immune function of the innateImmCluster subtypes, the GSVA analysis was performed and it was found that most of the immune-related pathways were significantly repressed in the cluster A group with poorer prognosis of innateImmCluster molecular subtypes compared with cluster B or cluster C with better prognosis in breast cancer patients, including cytokine-cytokine receptor interaction, T cell receptor signaling pathway, chemokine signaling pathway and B cell receptor signaling pathway (Figure 2C and Figure S3C). The GSVA results are consistent with the pathway enrichment analysis and suggested that the innateImmCluster molecular subtypes could be used for the immunophenotyping of breast cancer patients. To further examine these results, analysis for the immune infiltration of these three innateImmCluster molecular subtypes in breast cancer patients and found that the lower abundance of immune cell infiltration in cluster A innateImmCluster molecular subtypes than that in cluster B or cluster C innateImmCluster molecular subtypes in breast cancer patients (Figure 2D). Besides, the expression of those 15 innate immune-related prognostic signatures were identified and significantly differentially expressed among these three innateImmCluster molecular subtypes in breast cancer patients (Figure S3D).

### 3.3. Calculated the ICI Score to Forecast the Prognosis for Breast Cancer Patients

**Figure 3:**
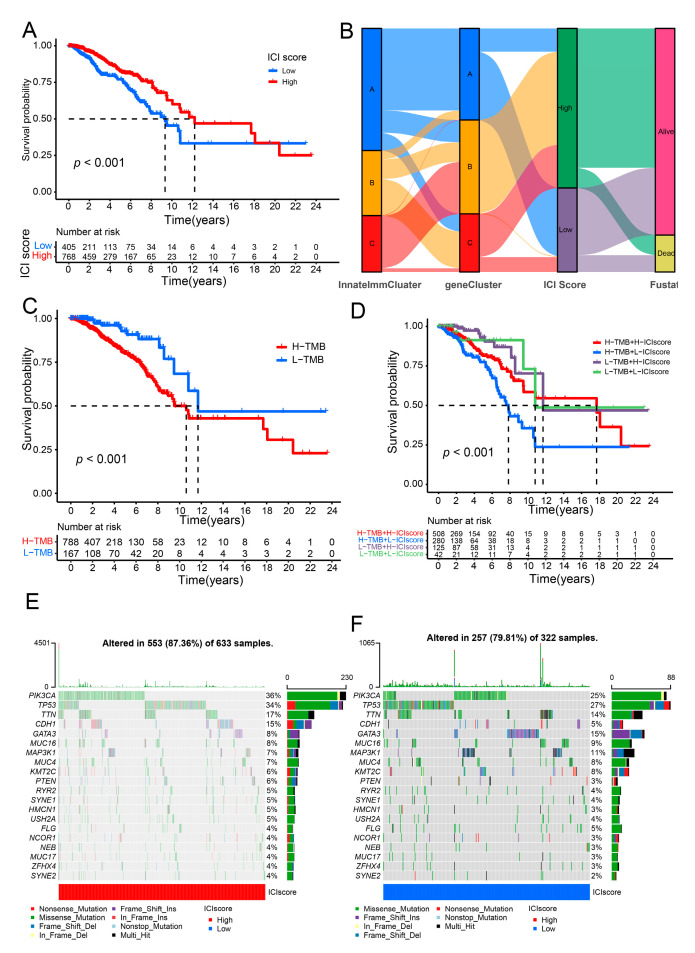
Constructed the ICI score model in breast cancer patients. (**A**) Comparison of the high ICI score level group and the low ICI score level group of overall survival of breast cancer patients through Kaplan–Meier survival analysis. (**B**) Prediction of the connection of innateImmCluster subtype, genecluster subtype, ICI score and the survival status of prognostic in breast cancer patients were shown via sankey plot. (**C**) Analysis of overall survival probability of breast cancer between the high TMB level group and the low TMB level group. (**D**) Combination of the ICI score and TMB level, the overall survival probability of breast cancer prognosis were analyzed. Comparison of the mutation rates of the top 20 genes between the high ICI score of breast cancer (**E**) and the low ICI score of breast cancer (**F**) through maftools.

To further explore the role of the innate signatures in tumor immunotherapy, we constructed the genecluster subtype of breast cancer via the expression levels of differentially expressed among innateImmCluster molecular subtypes samples through principal component analysis. And overall survival analysis showed that the survival probability was significantly differences among these three genecluster subtypes of breast cancer (Figure S4A). Then, we further identified the 97 prognostics signatures significantly correlated with the progress of breast cancer from differentially expressed genes among genecluster subtypes of breast cancer patients through univariate Cox regression analysis. And the innate cluster immune (ICI) score was calculated through the expression levels of these 97 prognostics signatures in breast cancer patients. To examine the effectiveness of ICI score, overall survival analysis was performed and found that the higher survival probability in the high ICI score group of breast cancer patients than that in the low ICI score group of breast cancer patients (Figure 3A). The connection of innateImmCluster subtype, genecluster subtype, ICI score and the status of prognostic in breast cancer patients were shown in the Figure 3B through R packages, ggalluvial. Recently research suggested that Tumor Mutational Burden (TMB) could be a new effective biomarker for cancer immunotherapy through the WGS and WES analysis in multiple cancer type. Thus, TMB analysis was performed for breast cancer patients. The overall survival analysis suggested that the high TMB levels group in breast cancer with poorer prognosis compared with the low TMB levels group in breast cancer patients (Figure 3C). And TMB analysis combined with ICI score in breast cancer shows that the high TMB level with high ICI score exhibited better prognosis and survival probability than that in the high TMB level with low ICI score in breast cancer patients. Consistently, the low TMB level with high ICI score exhibited better prognosis and survival probability than that in the low TMB level with low ICI score in breast cancer patients (Figure 3D). The mutational panorama and mutation rates of the top 20 genes in the high ICI score of breast cancer patients and the low ICI score of breast cancer patients were shown in Figure 3E,F. The correlation of immune cell infiltration and the ICI score were further analyzed and demonstrated that ICI score significantly positively correlated with most of the infiltration abundance of immune cells (Figure S4B). Then, the expression levels of these 15 innate immune-related signatures were detected and exhibited significantly differentially expressed among these three genecluster subtype samples of breast cancer (Figure S4C). Furthermore, the ICI score were exhibited significantly differences among the innateImmCluster subtypes or genecluster subtypes (Figure S4D,E). Besides, the role of the ICI score in the prognostic in breast cancer was further examined through the prognosis of the other clinical features in breast cancer patients, including stage status, T, M, age and N. The distribution of the ICI score in clinical characteristics of breast cancer patients were analyzed and shown in Figure S5A–F, including survival status, age, stage, T, M and N. The overall survival probability between the high ICI score and the low ICI score in breast cancer patients with clinical characteristics of Stage, T, N and M were analyzed and shown in Figure S6A–H. Consistently, the high ICI score with better prognosis in most of clinical feature’s subgroup in breast cancer patients compared with the low score groups in breast cancer patients. These results suggested that the ICI score may become a new biomarker for cancer immunotherapy in breast cancer.

### 3.4. Constructed the Risk Model for the Prognostic in Breast Cancer Patients

**Figure 4:**
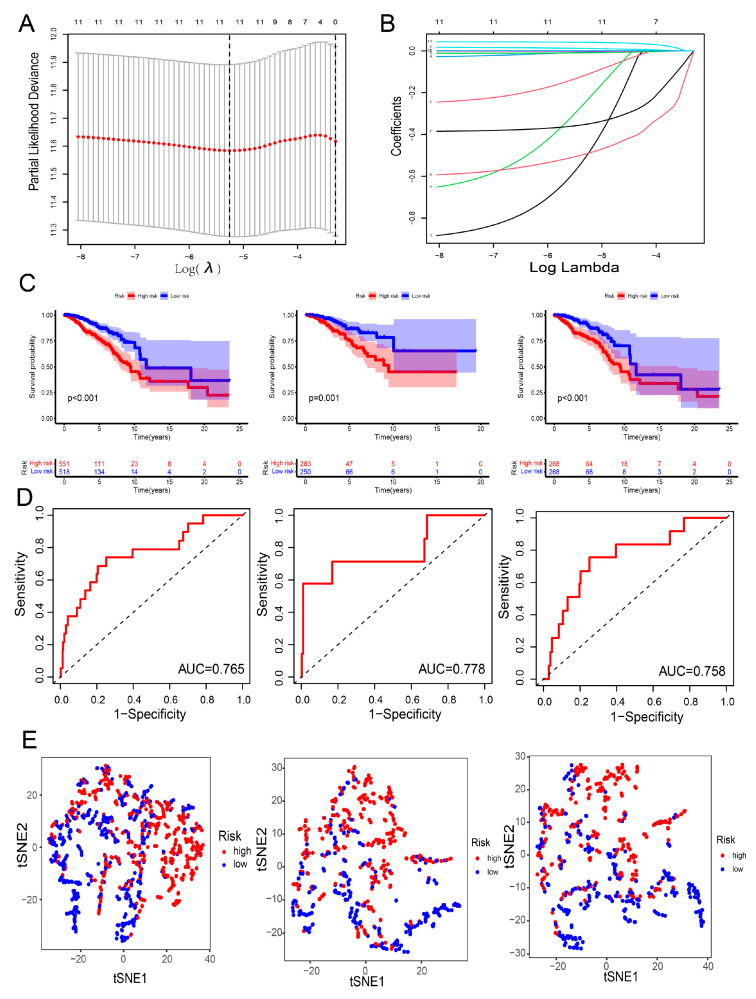
Identification of innate immune-related prognostic and constructed the risk model in breast cancer. The distribution of the partial likelihood deviation of the LASSO coefficient was shown in (**A**). (**B**) Exhibition of the LASSO coefficients of 11innate immune prognostic signatures. (**C**) Firstly, the sample of breast cancer patients were randomly divided into entire group, training group and a test group for prognostic analysis. Kaplan–Meier survival analysis between the high-risk group and the low-risk group of breast cancer patients of entire-set, training set and test set. (**D**) ROC curve of the risk prognostic model in breast cancer patients of entire-set, training set and test set. (**E**) The distribution of the risk score in breast cancer showing through PCA analysis.

Subsequently, the sample of breast cancer patients were randomly divided into a training group and a test group for further analysis. To further screened the innate immune checkpoint for cancer immunotherapy, the univariate cox analysis and the Lasso regression analysis were performed and 11 prognostic signatures were identified for constructed the risk model for the breast cancer prognosis in training group and test group (Figure 4A,B). And the risk score = coefficient(ELANE) × Exp(ELANE) + coefficient(NRG1) × Exp(NRG1) + coefficient(CLEC6A) × Exp(CLEC6A) + coefficient(IDO1) × Exp(IDO1) + coefficient(PLK1) × Exp(PLK1) + coefficient(CXCL9) × Exp(CXCL9) + coefficient(IL12B) × Exp(IL12B) + coefficient(CFB) × Exp(CFB) + coefficient(CRISP3) × Exp(CRISP3) + coefficient(IGHE) × Exp(IGHE). The overall survival analysis was performed between high-risk group and low risk group in entire-set, training set and test set. The results found that all low-risk with higher survival probability in all data sets compared with the high-risk groups (Figure 4C). The ROC analysis was performed and suggested that the risk model of prognosis in breast cancer patients with good predictive performance and reliability was shown in Figure 4D (AUC = 0.778 in training set; AUC = 0.758 in test set; AUC = 0.765 in entire set). Then the distribution of the risk score in breast cancer patients were shown in Figure 4E. Kaplan–Meier survival analysis showed that the high-risk group with lower survival probability and poorer prognosis than low-risk group in training set, test set and entire set in most of clinical characteristics group in breast cancer patients (Figure S7A–H). For instance, the high-risk score group with significant poorer survival prognosis than low-risk group in N1–3 phase patients, stage I–II phase patients, stage III–IV patients, T1–T2 phase patients and T3–T4 phase patients of breast cancer. Moreover, univariate and multivariate independent prognostic analysis further confirmed that these 11 innate immune prognostic signatures of the risk prognostic model can be independent of other clinical features for overall survival and outcome for breast cancer patients (Figure S8A,B).

### 3.5. Correlation of the Risk Model and Cancer Immunity

**Figure 5:**
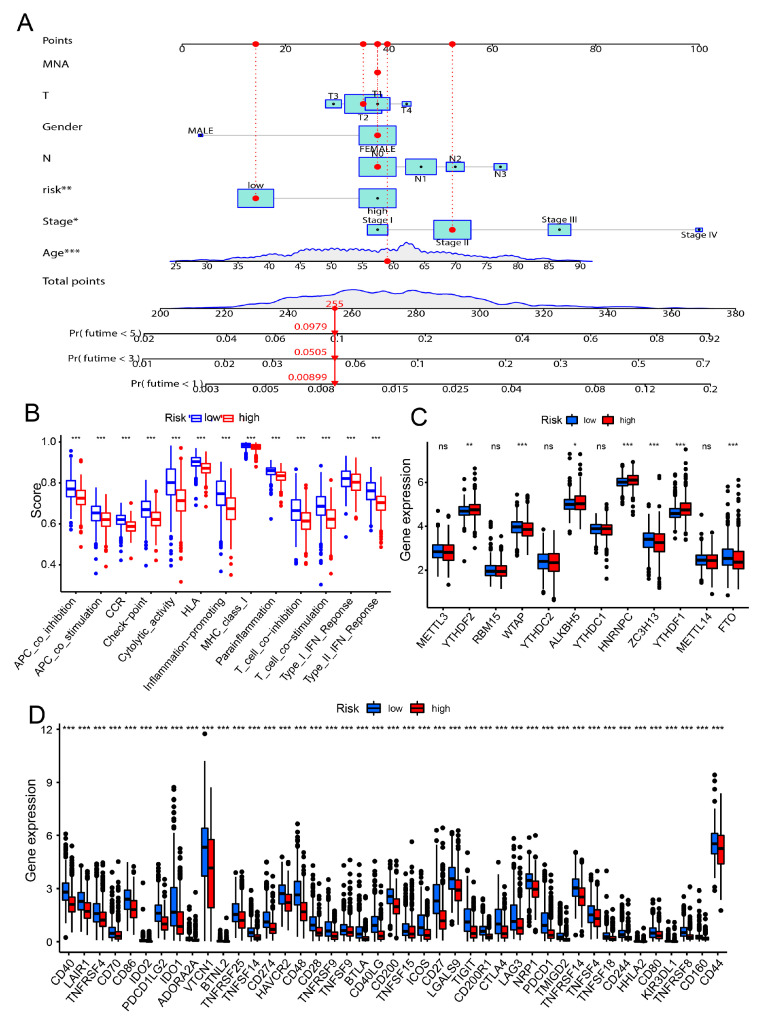
Correlation of the risk model and cancer immunity. (**A**) According to the risk model, prediction of the one-year, three-year and five-year overall survival of breast cancer patients through nomogram plot. (**B**) Comparison of the abundant of immune function pathway between the high-risk level group and the low-risk level group in breast cancer patients through GSVA analysis. (**C**) The expression levels of m6A readers and component of the m6A methyltransferase complex were showing between the high-risk level group and the low-risk level group in breast cancer patients. (**D**) Comparison of the expression levels of immune checkpoint in the high-risk level group and the low-risk level group of breast cancer patients. * *p* < 0.05, ** *p* < 0.01, *** *p* < 0.001, NS for not significant (*t*-test).

Subsequently, the nomogram plot was used to forecast the overall survival of breast cancer prognosis through the risk score and other independent prognostic factors of clinical characteristics in breast cancer at one year, three year and five years (Figure 5A). The heatmap showed that the expression levels of these 11 innate immune prognostic signatures between high-risk group and low risk-group were exhibited in breast cancer patients (Figure S9A). To further confirmed the role of the risk score in immune infiltration, the heatmap suggested that most of the abundant of immune cells were significantly suppressed in high-risk group compared with the low-risk group in breast cancer patients (Figure S9B). Besides, the pathway of immune-related were significantly repressed in the high-group compared with the low-group in breast cancer patients (Figure 5B). Recently, components of m6A readers and methyltransferase complex play the key role in cancer immunotherapy in multiple cancer. Thus, the expression of these m6A related factors were analyzed and it was found most of these factors were significantly differentially expressed between the high-risk group and the low-risk group in breast cancer patients (Figure 5C). Furthermore, we further analysis the correlation of risk score and immune checkpoint in breast cancer and demonstrated that most of key checkpoint were significantly differentially expressed between the two groups in breast cancer patients, including CD44, TIGIT, CTLA4, CD274, CD86 and CD80 (Figure 5D). 

### 3.6. Identification of CXCL9 as the Key Innate Immune-Related Prognostic Biomarker for Breast Cancer through Pan Cancer Analysis and Immune Infiltration Analysis

**Figure 6:**
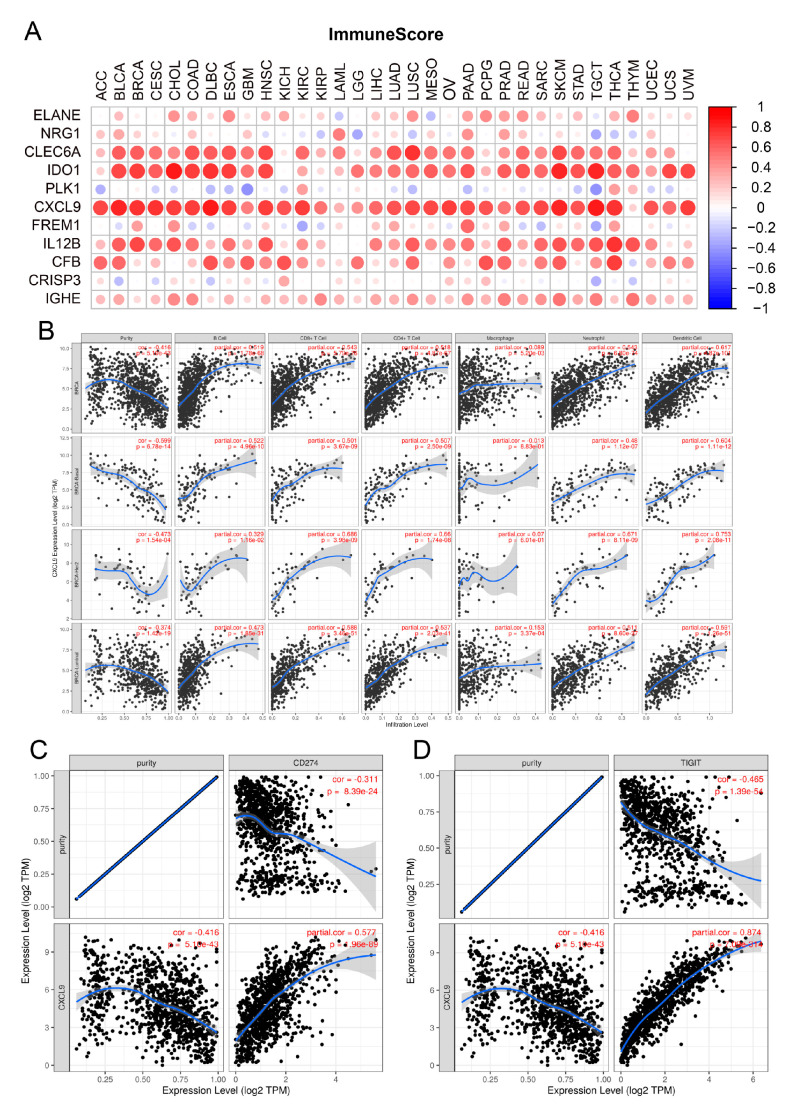
Immune cell infiltration analysis for innate prognostic biomarkers through transcriptome profile. Comparison of the correlation of the expression of 11 innate immune prognostic biomarkers and the levels of immune score (**A**) of multiple cancer patients through the transcriptome profile. (**B**) Analysis for the correlation of the expression of CXCL9 and the abundance of immune cell infiltration in breast cancer patients through TIMER database. The expression of CXCL9 exhibited significant negative correlation with the key checkpoint CD274 (**C**) and TIGIT (**D**) in breast cancer.

Pan cancer analysis was performed and it was found that only 4 out of these 11 innate immune prognostic biomarkers were significantly different between the high expression level and low expression level in the prognosis of multiple cancer type, including breast cancer and lung cancer (Figure S10A–D). Then, immune infiltration analysis was performed through ESTIMATE and CIBERSORT methods. The results suggested that CXCL9 is one of the most correlations of abundant of immune cell infiltration than other ten innate immune prognostic biomarkers in multiple cancer types (Figure 6A and Figure S11A,B). For instance, the expression of CXCL9 was significantly positively correlated with the most of the immune score and most of the immune cell infiltration. Consistently, the expression of CXCL9 was significantly negatively correlated with the tumor purity in multiple cancer types. Moreover, the expression level of CXCL9 significantly increased in triple negative breast cancer patients compared with the HER2-enriched or ER-positive breast cancer patients (Figure S11C). To further examine these results, the correlation of the expression of CXCL9 and immune cell infiltration were analyzed through TIMER database. According to the results, expression of CXCL9 was significantly correlated with immune cell infiltration in breast cancer patients and subtype patients, including basal-like, luminal-like and HER2 enriched (Figure 6B). Moreover, the correlation of the expression of CXCL9 and the two key immune checkpoints were analyzed and CXCL9 exhibited significantly negative correlation with immune checkpoint (CD274 and TIGIT) (Figure 6C,D). Taken together, CXCL9 could be a new innate immune checkpoint for breast cancer therapy and immunotherapy.

### 3.7. Identification of Small Molecular Drug for Target CXCL9 through Molecular Docking

**Figure 7:**
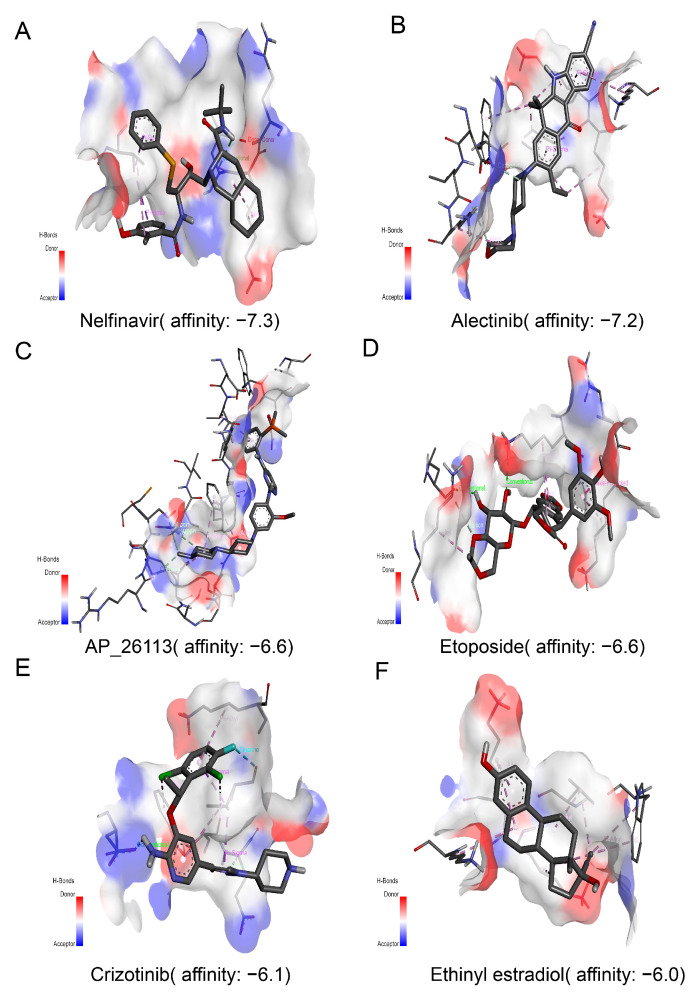
Molecular docking analysis for identification of small molecular drug for target CXCL9. (**A**–**F**) According the binding affinity between the small molecular drug and CXCL9, three small molecular drugs were selected with high affinity to bind CXCL9 through Autodock-vina tools, including alectinib: affinity = −7.2, nelfinavir: affinity = −7.3 and etoposide: affinity = −6.6.

Furthermore, we further performed molecular docking analysis to identify small molecular drug of target CXCL9 for breast cancer immunotherapy. Firstly, we analyze for the correlation of the expression level of CXCL9 and small molecular target drug through the drug sensitivity analysis via CELLMINER database. In total, we have identified 16 small molecular target drugs for CXCL9. The 3D structure of these 16 small molecular target drugs were downloaded from PubChem database. Then, molecular docking analysis was performed to further screen the effector drug bind to the protein structure of CXCL9 via AutoDock-vina tools. The docking results were visualization through discovery studio and shown in Figure S12A–L. And the top six small molecular drugs were selected for further research. The binding sites were shown in Figure 7A–F and Figure S13A–F. Among these small molecular target drugs, the top three drugs of target CXCL9 were selected, including alectinib, nelfinavir and etoposide (alectinib: affinity = −7.2; nelfinavir: affinity = −7.3; etoposide: affinity = −6.6). These results suggested that CXCL9 significantly correlated with the prognostic of breast cancer and immune cell infiltration and could be innate immune checkpoint for breast cancer immunotherapy.

## 4. Discussion

Breast cancer is the most common female malignant tumor in the world [1]. Recently, more and more research evidence demonstrated that tumor immunity will become the most powerful weapon to overcome the treatment of all cancer types. The importance of innate and adaptive immune response in cancer were gradually paid attention to by researchers [12,20,30]. And recently, related research keeps pouring out. Although, the molecular mechanism of innate immune in breast cancer is still unknown. The main purpose of this study is to identify innate immune-related prognostic signatures as checkpoint for immunotherapy of breast cancer patients. In this study, based-on the transcriptome profiles analysis, univariate Cox regression analysis and overall survival analysis for innate immune related genes in breast cancer patients, 15 innate immune-related prognostic signatures were identified and selected for further analysis, including CXCL9, TP63, IL12B, IL33 and so on. Interestingly, most of these selected prognostic biomarkers play the key roles in the prognosis of multiple cancer, including lung cancer, liver cancer and breast cancer. Therefore, these selected out 15 innate immune-related signatures may become series of effective target for breast cancer therapy.

Recently, immunotherapy has significantly prolonged the overall survival prognosis of multiple cancer types [10,19,26]. And most evidence showed that subtype of immunophenotyping of multiple cancer patients could provide more effective reference information for cancer immunotherapy [43]. In addition, the effect of immunotherapy in cancer patients mostly benefits from the innate immune response. Most of innate immune-related pathways and cells contributed to cancer immunotherapy. For instance, type I IFN pathway [44], TLR signaling pathway [45] and chemokine pathway [46]. To further screen checkpoint of innate immune response, the three subtype of innate immune in breast cancer patients through the expression levels of these 15 identified innate immune prognostic signatures and termed as innateImmCluster molecular subtype. Overall survival probability significantly differs among these three innateImmCluster subtype of breast cancer. And the most of that immune related response pathways were significantly repressed in cluster A with poorer prognosis compared with cluster B or cluster C with better prognosis in breast cancer patients through GSVA analysis, GO and pathway enrichment analysis. In addition, the abundance of immune cell infiltration also revealed cluster A of patients with the lower the abundant of immune cell infiltration than that in cluster B or cluster C. These results suggested that the cluster A of innateImmCluster molecular subtype with poorer prognosis and significantly suppressed the level of immune infiltration in breast cancer could closely reflect the immune infiltration status of cancer patients.

Subsequently, the ICI risk model was calculated through the transcriptome profiles of differentially expressed genes among the three innateImmCluster molecular subtype of breast cancer patients. It’s noted that the high level of the ICI score exhibited better prognosis and survival probability compared with the low level of the ICI score group in breast cancer patients. Recent research suggested that tumor mutational burden (TMB) become a new marker of cancer prognosis and to predict the effective of cancer immunotherapy [47]. Generally, the high TMB level groups had poorer prognosis and survival probability than those in the low TMB level groups in breast cancer patients. Interestingly, while the high level of ICI score in high TMB group patients showed significantly improvement, the overall survival status compared with the low level of ICI score in high TMB groups in breast cancer. Consistently, the similar results were exhibited in the low TMB group combined with the ICI score in breast cancer. Thus, these results revealed that the ICI score could become a new biomarker for prediction of breast cancer prognosis. To further identify the key innate immune signatures as checkpoints for breast cancer immunotherapy, based on the expression level of those 15 innate immune related signatures, the risk model was constructed through the univariate cox analysis and the Lasso regression analysis and identified 11 prognostic biomarkers in breast cancer, including ELANE, NRG1, CLEC6A, IDO1, PLK1, CXCL9, FREM1, IL12B, CFB, CRISP3 and IGHE. The overall survival and univariate and multivariate cox analysis further confirmed the reliability and effectiveness of the risk model in breast cancer prognosis. Moreover, the correlation of the risk model and the abundant of immune cell infiltration were analyzed and suggested that most of the immune cell exhibited significantly suppressed the abundant of immune infiltration in the high-risk group compared with the low-risk group in breast cancer patients. In addition, the expression of m6A readers, ferroptosis-related genes and immune checkpoint molecule were all revealed significantly differentially expressed between the high-risk level of group and the low-risk of group in breast cancer patients. Taken together, the ICI score and the risk score may become a new biomarker for cancer immunotherapy in breast cancer, and these 11 innate immune prognostic signatures could be an effective checkpoint for checkpoint immunotherapy for breast cancer patients.

Pan cancer analysis and the tumor immune microenvironment analysis further suggested that CXCL9 has greater importance in cancer immunotherapy than other prognostic biomarkers. Recent related studies suggested depletion of CXCL9 significantly affected T cell infiltration and the anti-PD1 immunotherapy. Therefore, we focus on the CXCL9 for further study. With the progress of computer aid drug design, small molecular target drug screening was widely used for cancer therapy, including immunotherapy. Therefore, we identified small molecular target drug for CXCL9 through molecular docking. And 12 small molecular drugs with high affinity to bind CXCL9. These drug all FDA approves Drugs and collected form CELLMINER database.

In summary, in this study the ICI score and risk score may become a new biomarker for cancer immunotherapy in breast cancer. CXCL9 can be innate immune checkpoint for cancer immunotherapy. However, some limitations still exist. Firstly, the ICI score model and the risk model were constructed only according to the transcriptome expression level of innate immune signatures from public database TCGA and GEO. Secondly, experimental research verification and a large number of clinical phenotypic verification are still lacking. Finally, whether CXCL9 can be determined as a new prognostic checkpoint for breast cancer immunotherapy remains to be further verified.

## Data Availability

All data sources and analysis methods that support the findings of this study are available in the methods, supplementary materials, The Cancer Genome Atlas (TCGA) (https://portal.gdc.cancer.gov/, accessed on 14 April 2021), ucsc-xena (https://xenabrowser.net/datapages/, accessed on 14 April 2021), Gene Expression Omnibus (GEO) (https://www.ncbi.nlm.nih.gov/geo/, accessed on 14 April 2021), CELLMINER public database (https://discover.nci.nih.gov/cellminer/, accessed on 25 August 2021) and PubChem (https://pubchem.ncbi.nlm.nih.gov/, accessed on 30 August 2021).

## Supplementary Materials

The following are available online at https://www.mdpi.com/article/10.3390/genes13010088/s1, Figures S1–S13, Figure S1: Identification and screening of differentially expressed innate immune-related signatures; Figure S2: Screening of innate immune-related prognostic signatures in breast cancer; Figure S3: Construction of three subtype of breast cancer through the level of innate immune-related prognostic signatures; Figure S4: Constructed the ICI score model in breast cancer patients; Figure S5: The distribution of ICI score in multiple clinical characteristics of breast cancer patients; Figure S6: Correlation analysis for between the overall survival probability and the ICI score in clinical characteristics of breast cancer patients; Figure S7: Correlation analysis for the overall survival probability and the risk score in clinical characteristics of breast cancer patients; Figure S8: Univariate and Multivariate independent prognostic analysis; Figure S9: Correlation analysis of between the risk model and the immune infiltration in breast cancer; Figure S10: Pan cancer analysis for innate prognostic biomarkers through transcriptome profile; Figure S11: Analysis for the correlation of the expression of innate immune prognostic signatures and immune infiltration in breast cancer; Figure S12: Molecular docking analysis for identification of small molecular drug for target CXCL9; Figure S13: Top 6 small molecular drug for target CXCL9 through molecular docking analysis.
