# Supplementary material for: Comprehensive Analysis of Innate Immunophenotyping Based on Immune Score Predicting Immune Alterations and Prognosis in Breast Cancer Patients
Source: Genes (Basel). 2021 Dec 29;13(1):88. doi: 10.3390/genes13010088 (PMC8774675; doi:10.3390/genes13010088)
Supplement: Supplementary file 1 [file genes-13-00088-s001.zip › genes-1519663-supplementary.pdf]

genes MDPI

Additional information for

Comprehensive analysis of immune subtyping based-on immune score predicting immune alterations and prognosis in breast cancer patients

Weiguang Liu, Lingling Xia, Zhengmiao Xia, Liming Chen.

Correspondence to: chenliming1981@njnu.edu.cn

This Additional information file includes:

Figure S1 to S13
Figure S1: Identification and screening of differentially expressed innate immune-related signatures;
Figure S2: Screening of innate immune-related prognostic signatures in breast cancer;
Figure S3: Construction of three subtype of breast cancer through the level of innate immune-related prognostic signatures;
Figure S4: Constructed the ICI score model in breast cancer patients;
Figure S5: The distribution of ICI score in multiple clinical characteristics of breast cancer patients;
Figure S6: Correlation analysis for between the overall survival probability and the ICI score in clinical characteristics of breast cancer patients;
Figure S7: Correlation analysis for the overall survival probability and the risk score in clinical characteristics of breast cancer patients;
Figure S8 Univariate and Multivariate independent prognostic analysis;
Figure S9: Correlation analysis of between the risk model and the immune infiltration in breast cancer;
Figure S10: Pan cancer analysis for innate prognostic biomarkers through transcriptome profile;
Figure S11: Analysis for the correlation of the expression of innate immune prognostic signatures and immune infiltration in breast cancer;
Figure S12: Molecular docking analysis for identification of small molecular drug for target CXCL9;
Figure S13: Top 6 small molecular drug for target CXCL9 through molecular docking analysis.

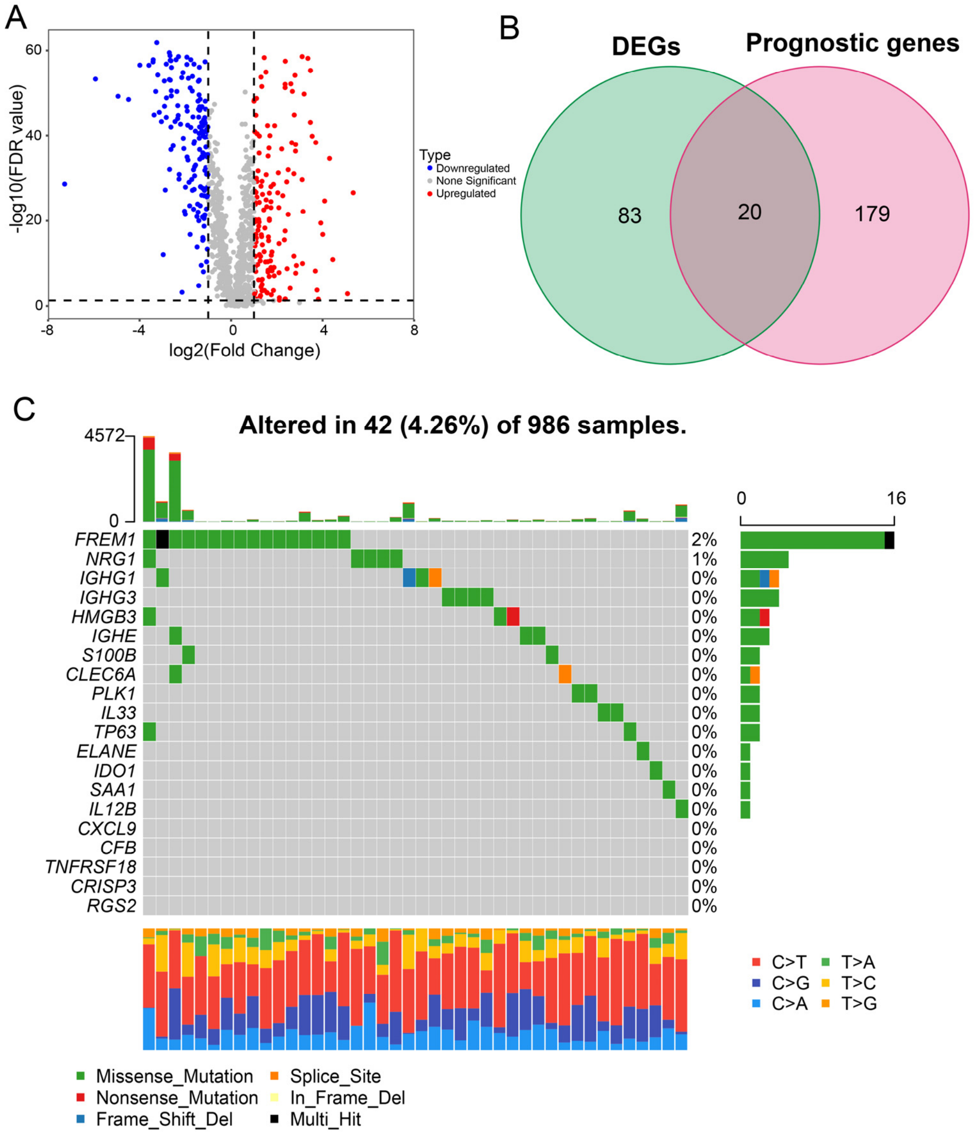

Figure S1 Identification of differentially expressed innate immune-related signatures. (**A**) Volcano plot showing the differentially expressed innate immune-related genes berween breast cancer group and normal group (|logFC|>1; FDR<0.05). (**B**) Venn plot showing the overlap of the differentially expressed innate immune-related genes and prognostic genes in breast cancer patients. (**C**) The mutation rates of innate immune related signatures in breast cancer through maftools.

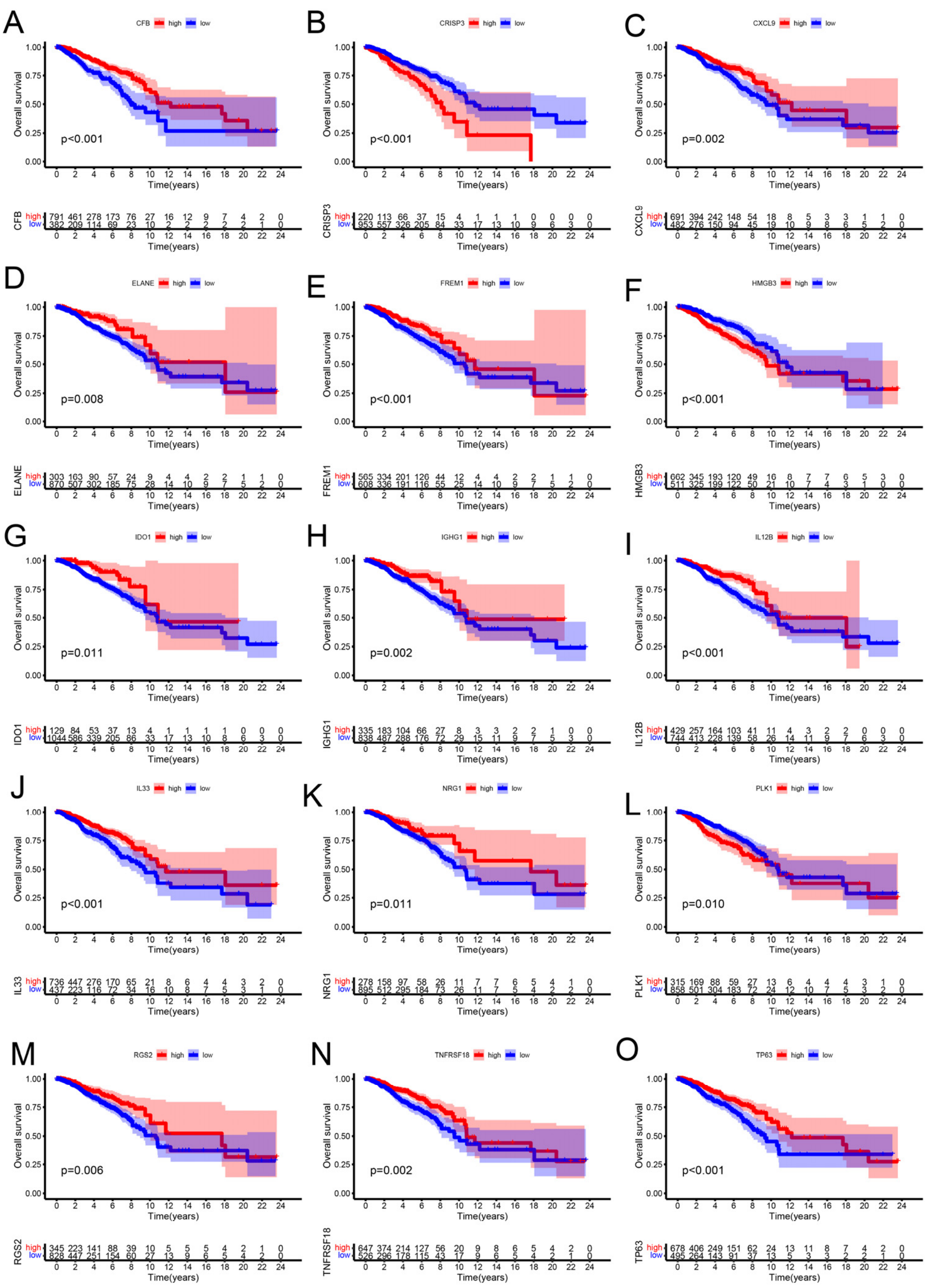

Figure S2 Screening of innate immune-related prognostic signatures in breast cancer. (**A**)-(**O**) Overall survival analysis for innate immune-related prognostic signatures in breast cancer prognosis via the gene transcription profiles. And 15 innate immune-related prognostic signatures were identified and significantly correlated with the overall survival of breast cancer patients.

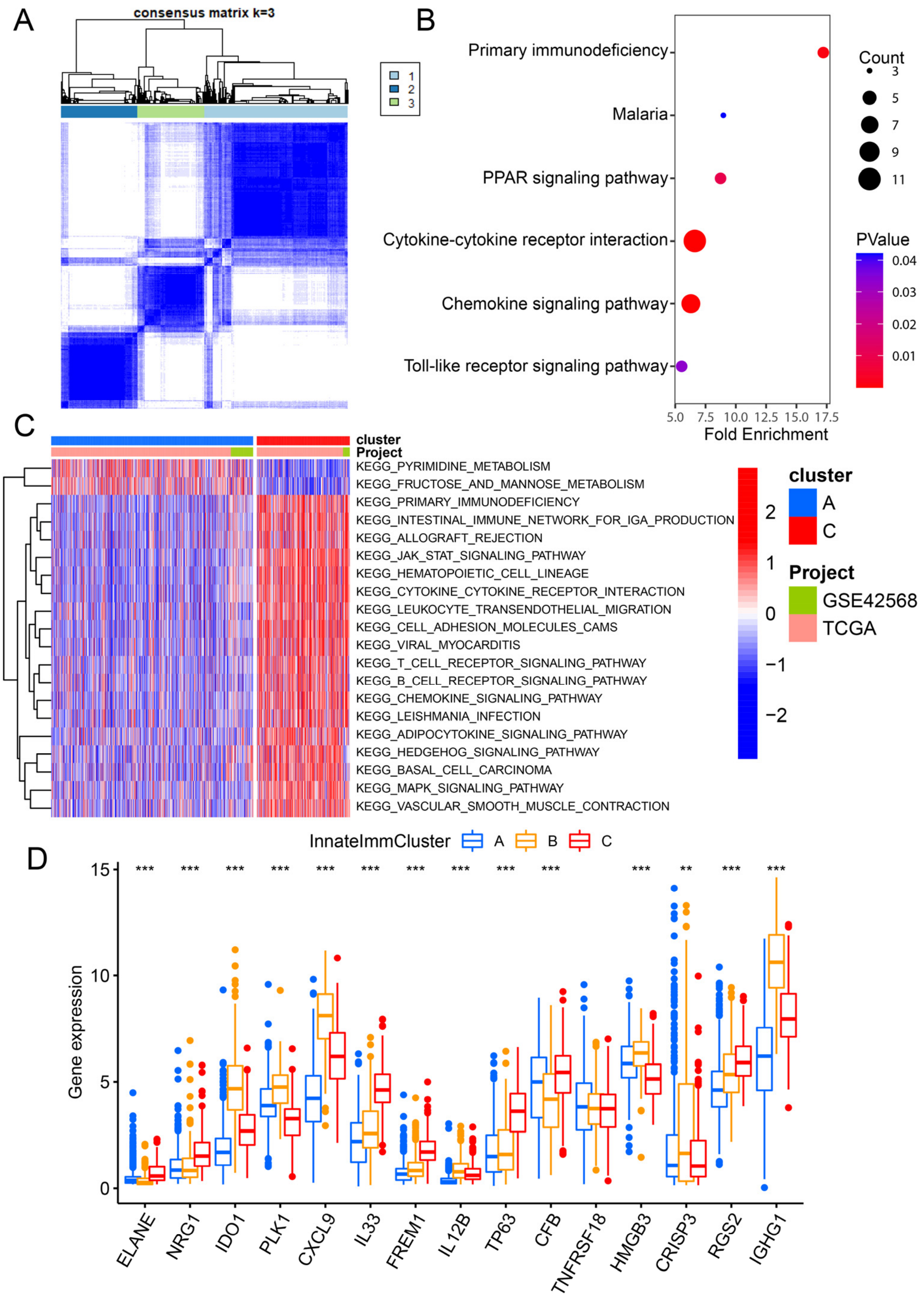

Figure S3 Construction of three subtype of breast cancer through the level of innate immune-related prognostic signatures. (**A**) Consensus matrix heatmap showing that the three cluster of InnateImmCluster molecular subtype in breast cancer were constructed. (**B**) KEGG enrichment pathway analysis for differentially expressed genes among the three cluster of InnateImmCluster molecular subtype in breast cancer. (**C**) GSVA analysis revealed that most of the immune response related pathway were significantly repressed in cluster A of InnateImmCluster molecular subtype in breast cancer compared with cluster C. (**D**) The expression levels of innate immune-related

signatures among these three clusters of InnateImmCluster molecular subtype in breast cancer patients were shown via boxplot.

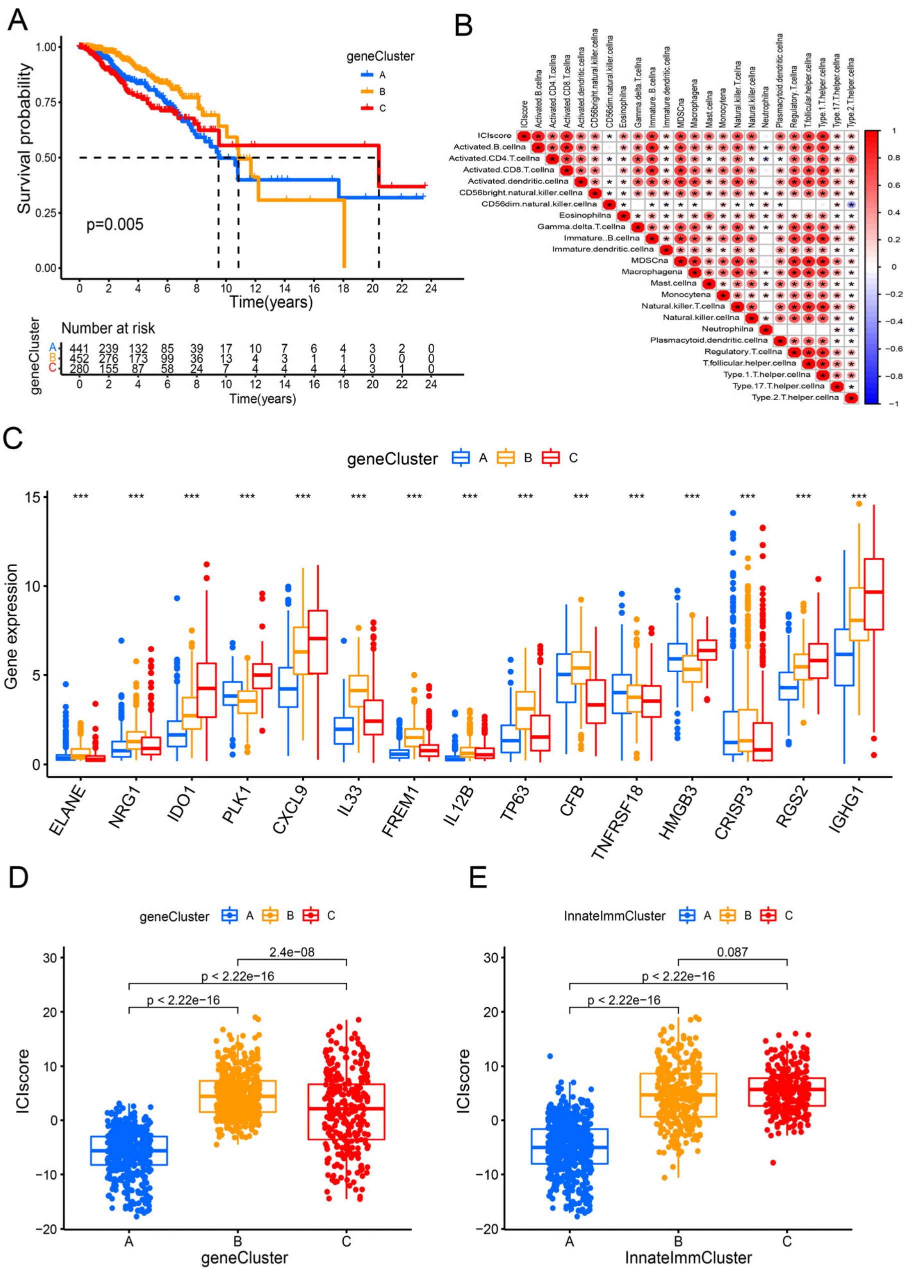

Figure S4 Constructed the ICI score model in breast cancer patients. (**A**) Constructed three cluster of genecluster subtype in breast cancer. And analysis for overall survival probability among three genecluster subtype of breast cancer patients. (**B**) Correlation of the ICI score and the abundant of immune cells infiltration in breast cancer. (**C**) Boxplot showing the expression level of innate immune related signatures among three cluster of genecluster subtype and in breast cancer. (**D**) and (**E**) Comparison of the ICI score among three cluster of genecluster and innateImmCluster subtype in breast cancer through boxplot.

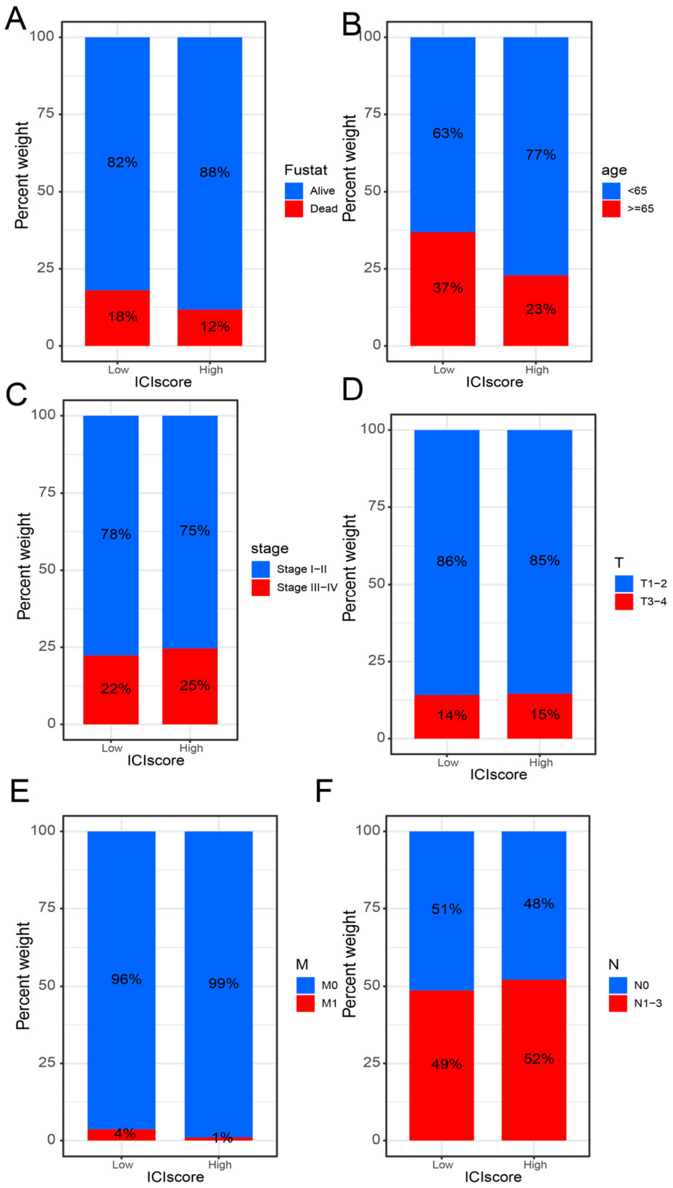

Figure S5 The distribution of ICI score in multiple clinical characteristics of breast cancer patients. (**A**)-(**F**) Quantitative analysis for the proportion of clinical characteristics in the high ICI score and the low ICI score in breast cancer patients, including survival status, age, stage, T, M and N.

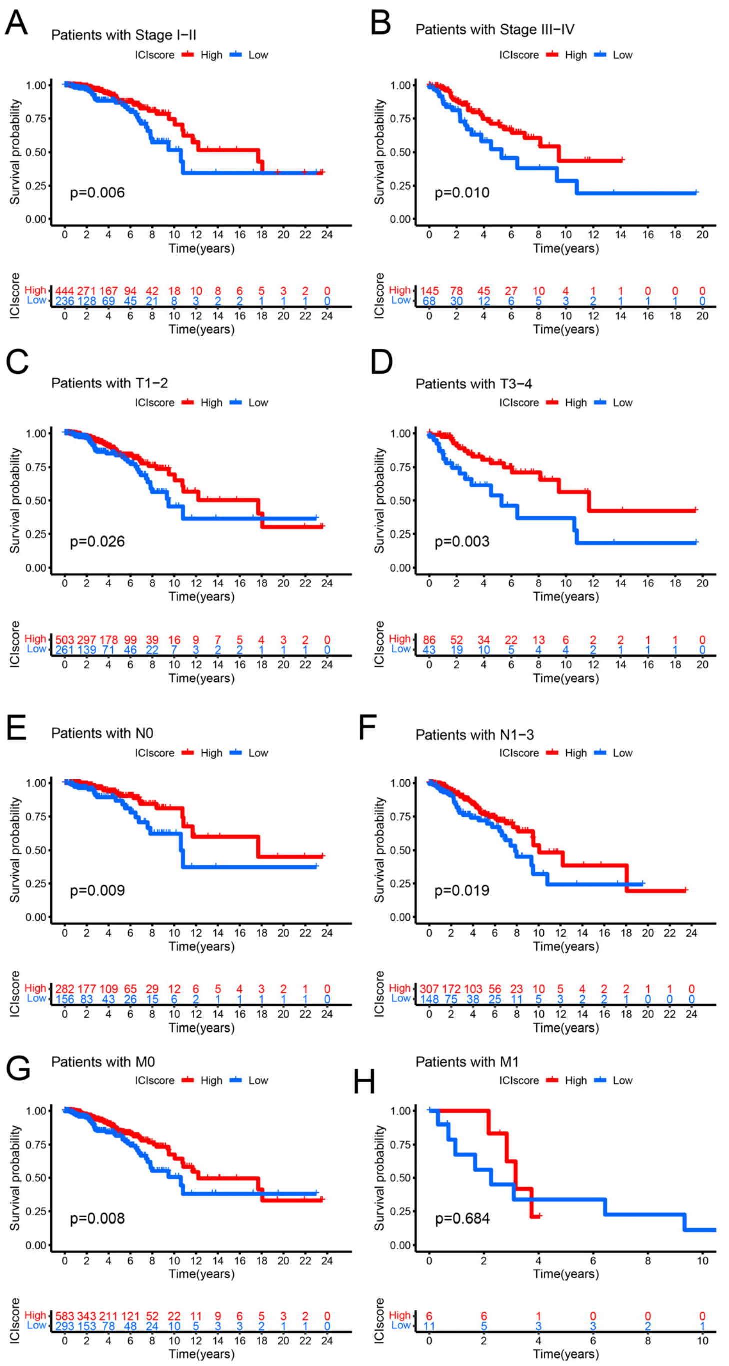

Figure S6 Correlation analysis for between the overall survival probability and the ICI score in clinical characteristics of breast cancer patients. (**A**)-(**H**) Analysis for the overall survival probability in the high ICI score and the low ICI score in breast cancer patients with clinical characteristics of Stage, T, N and M.

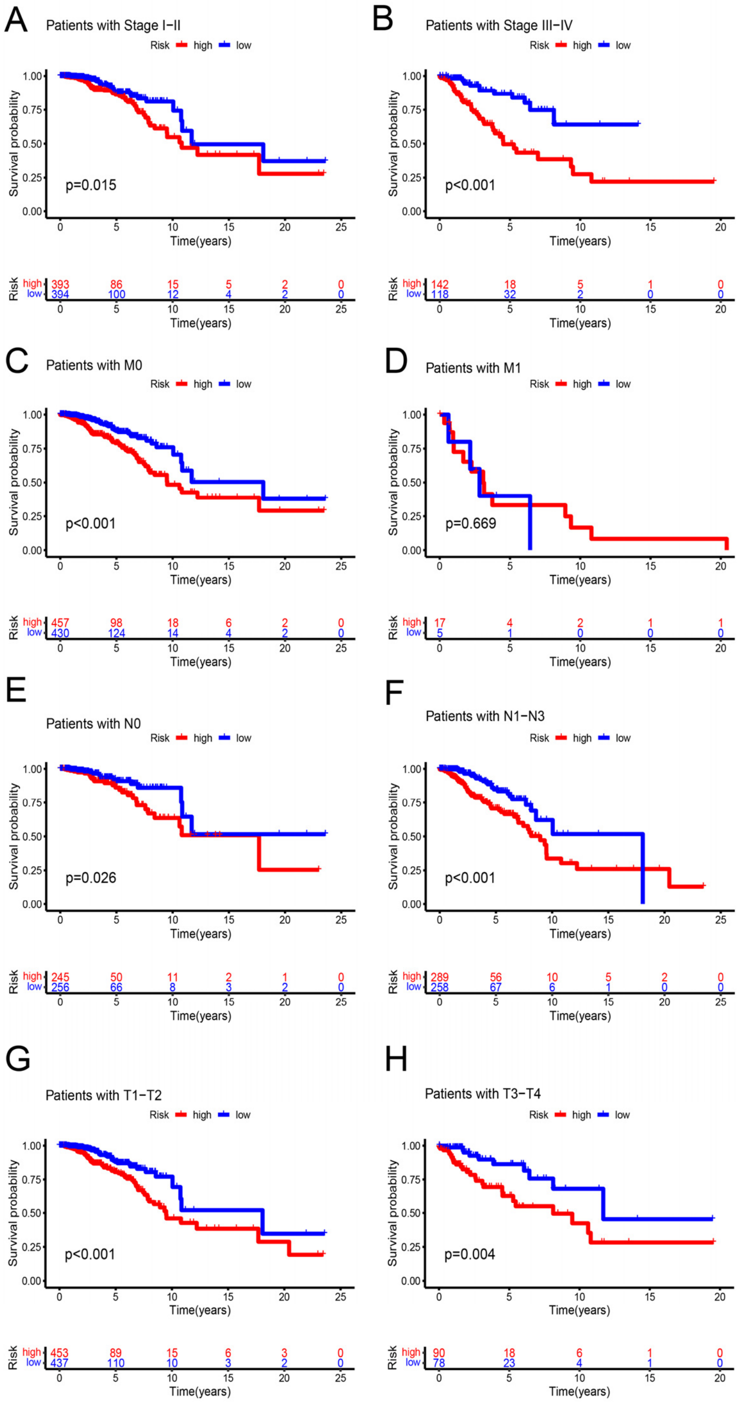

Figure S7 Correlation analysis for between the overall survival probability and the risk score in clinical characteristics of breast cancer patients. (**A**)-(**H**) Analysis for the overall survival probability in the high ICI score and the low ICI score in breast cancer patients with clinical characteristics of Stage, T, N and M.

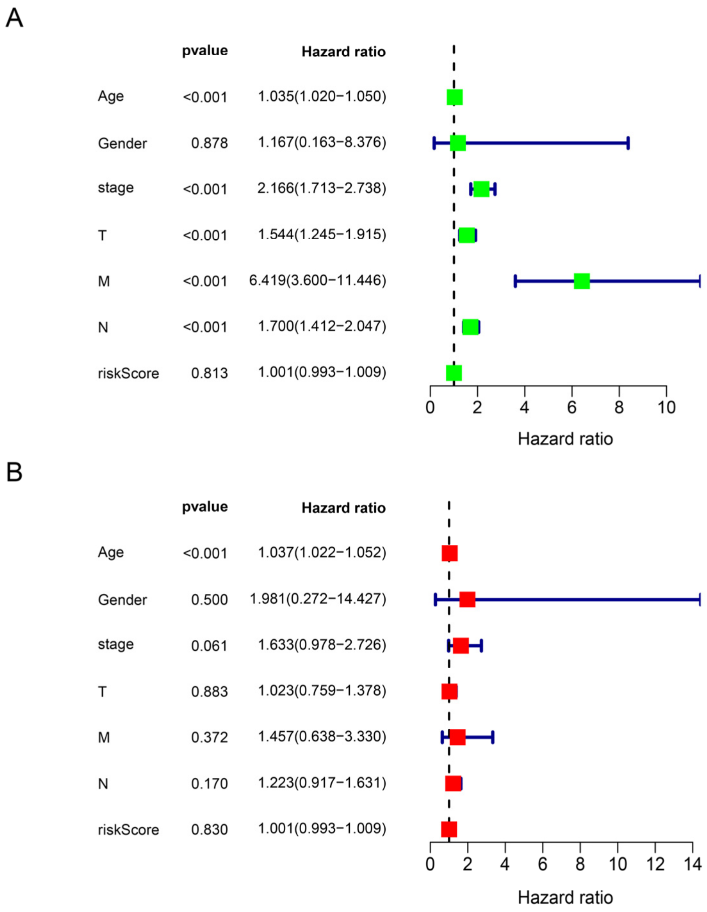

Figure S8 Univariate and Multivariate independent prognostic analysis. (A) Exhibition of univariate Cox analysis in breast cancer through forest map. (B) Exhibition of multivariate Cox analysis in breast cancer through forest map.

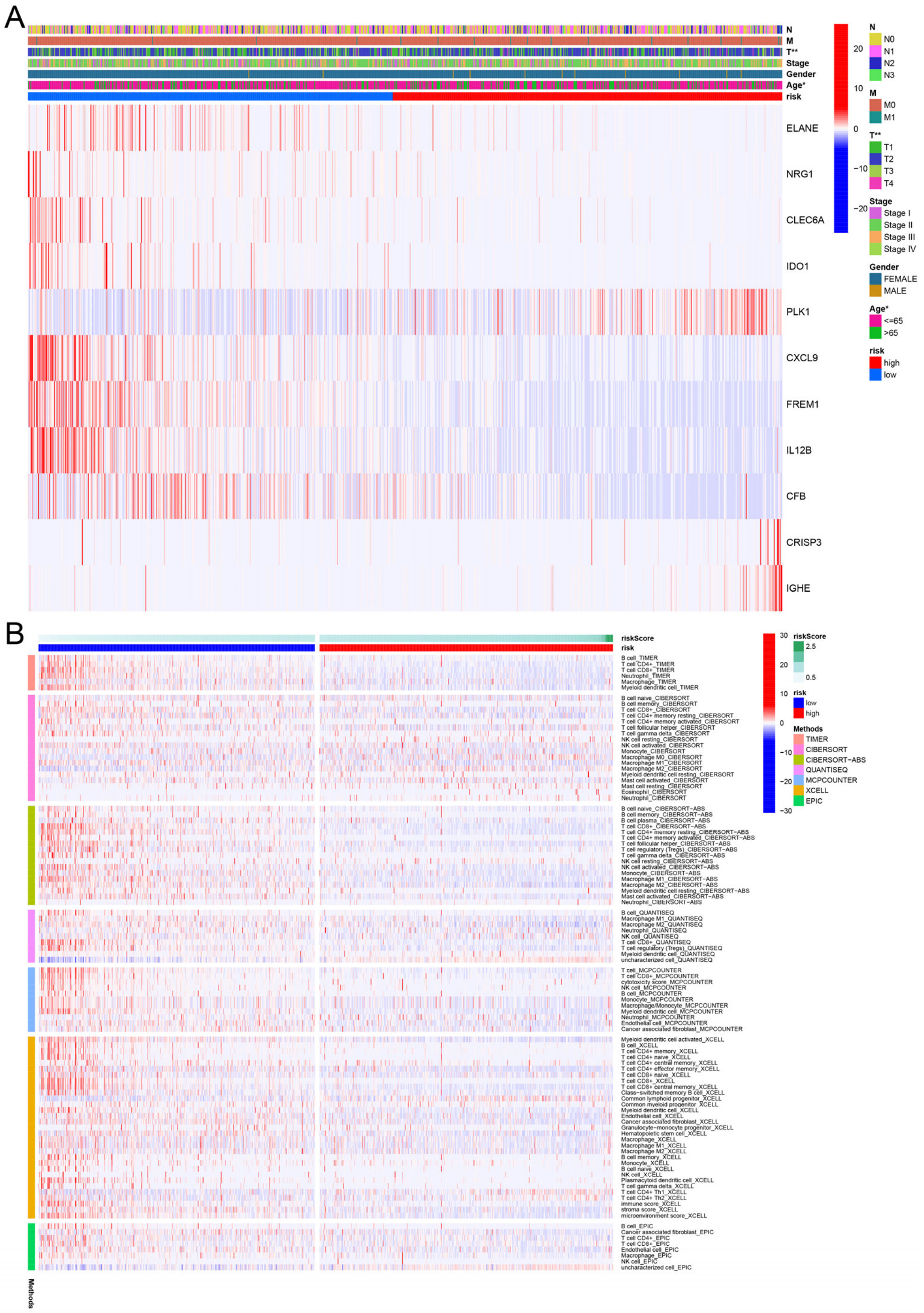

Figure S9 Correlation analysis of between the risk model and the immune infiltration in breast cancer. (**A**) The heatmap plot showing the expression level of CXCL9 between the high-risk group and the low-risk group of breast cancer patients. (B) The abundance of immune infiltration between the high-risk group and the low-risk group of breast cancer patients were shown via heatmap.

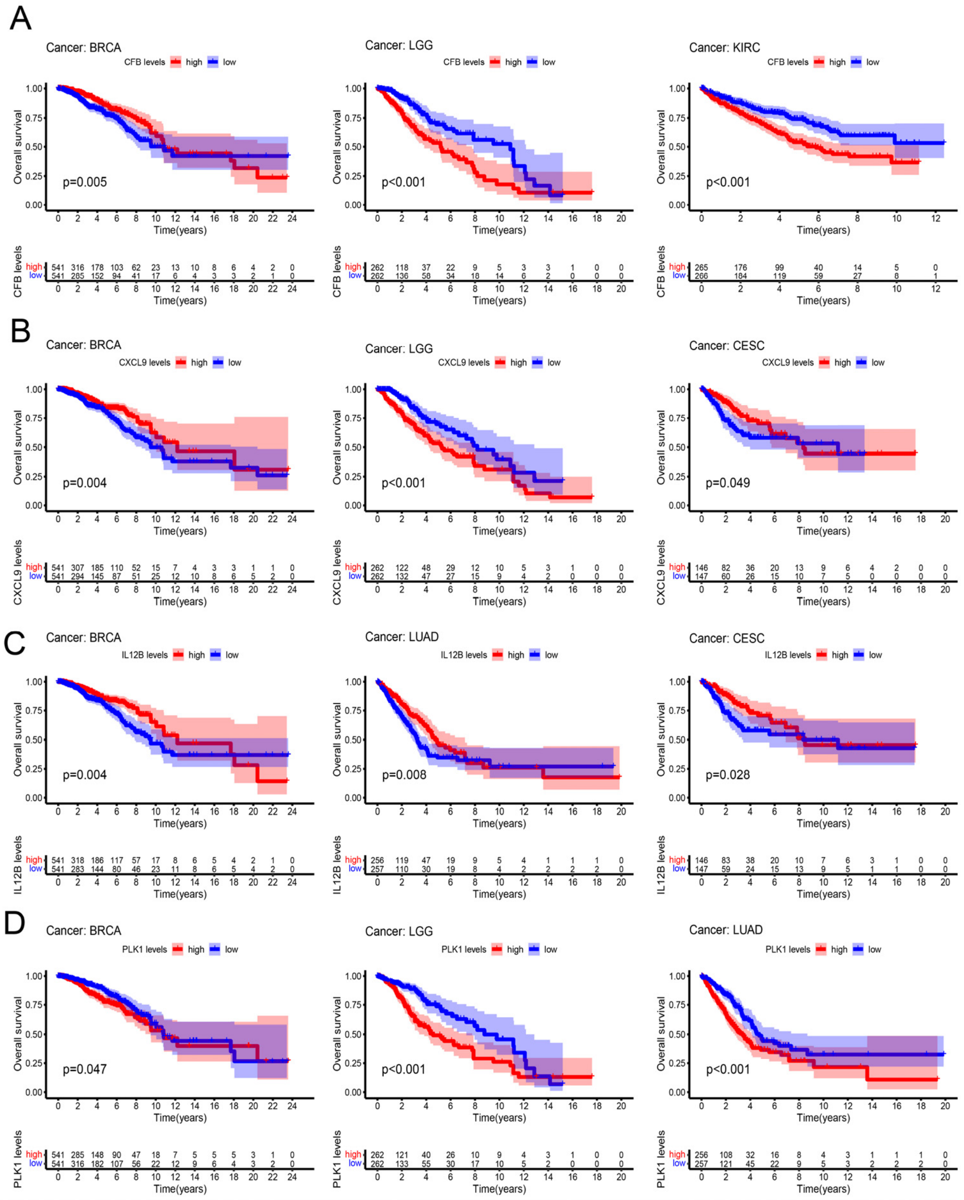

Figure S10 Pan cancer analysis for innate prognostic biomarkers through transcriptome profile. (**A-D**) Based-on the expression level of these innate immune prognostic biomarkers, the sample of multiple cancer type patients were divided into the high expression level and low expression level group. Analysis for the overall survival probability between the high-expression level group and the low-expression level group in multiple cancer type patients. And only 4 out of 11 innate immune prognostic biomarkers were selected, including CXCL9, CFB, IL12B and PLK1.

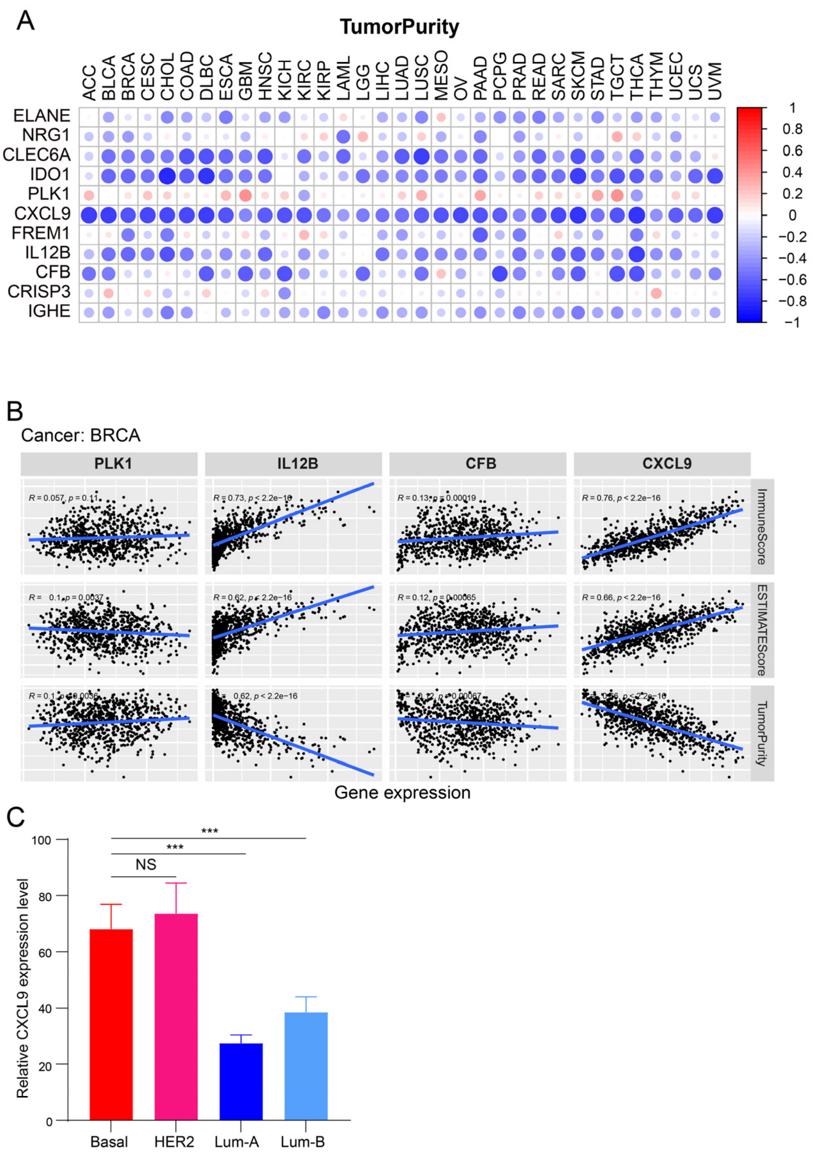

Figure S11 Analysis for the correlation of the expression of innate immune prognostic signatures and immune infiltration in breast cancer. (**A**) Comparison of the correlation of the expression of 11 innate immune prognostic biomarkers and the levels of tumor purity of multiple cancer patients through the transcriptome profile. (**B**) Analysis for the correlation of the expression of four key innate immune prognostic signatures and immune infiltration in breast cancer patients. The expression level of CXCL9 in subtype of breast cancer patients were shown in (**C**), including Basal, HER2-enriched, Luminal-A and Luminal-B.

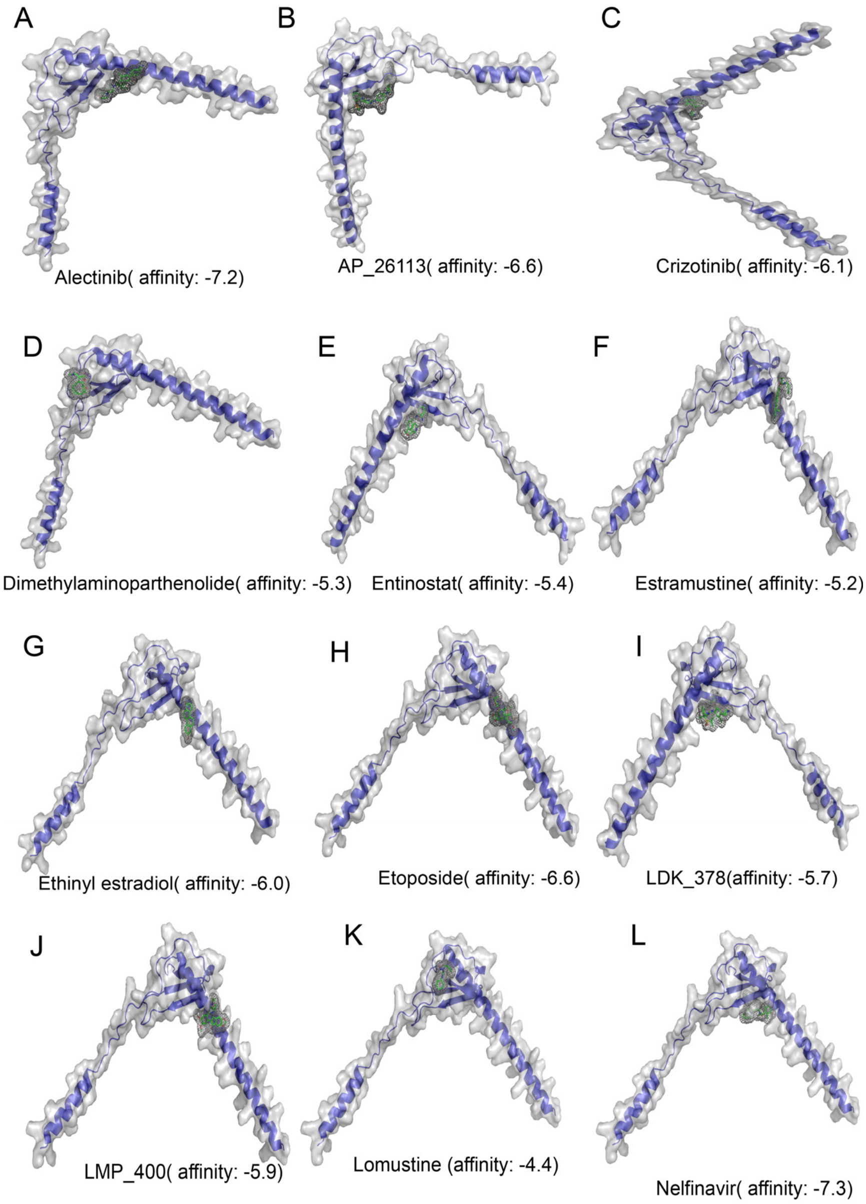

Figure S12 Molecular docking analysis for identification of small molecular drug for target CXCL9. (**A**-**L**) According the binding affinity between the small molecular drug and CXCL9, three small molecular drugs were selected with high affinity to bind CXCL9 through Autodock-vina tools, including alectinib: affinity = -7.2, nelfinavir: affinity = -7.3 and etoposide: affinity = -6.6.

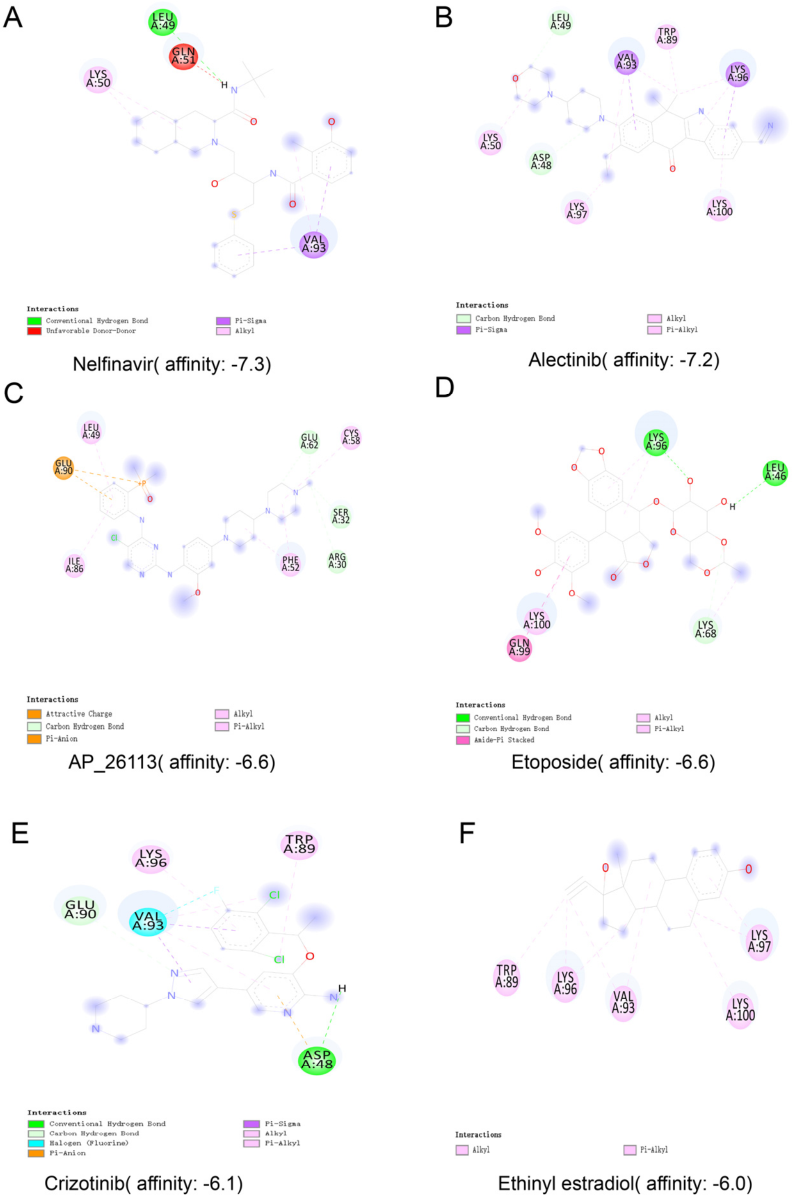

Figure S13 Top 6 small molecular drug for target CXCL9 through molecular docking analysis. (**A**-**F**) The binding sites beween the CXCL9 and six small molecular drugs were shown via the 2D diagram.
